# Patient Outcomes After Resection of Pigmented Villonodular Synovitis in Patients in King Abdulaziz Medical City

**DOI:** 10.7759/cureus.68248

**Published:** 2024-08-30

**Authors:** Wazzan Aljuhani, Amal Alamri, Byan Altorbak, Jawad Alabbasi, Faris Ahmed

**Affiliations:** 1 Department of Surgery, Ministry of the National Guard - Health Affairs, King Abdullah International Medical Research Center, King Saud Bin Abdulaziz University for Health Sciences, Riyadh, SAU; 2 Department of Surgery, Ministry of the National Guard – Health Affairs, King Abdullah International Medical Research Center, King Saud Bin Abdulaziz University for Health Sciences, Riyadh, SAU; 3 Department of Surgery, Ministry of the National Guard – Health Affairs, King Abdullah International Medical Research Center, King Saud Bin Abdulaziz University for Health Sciences, Riyadh, Saudi Arabia, Riyadh, SAU; 4 Collage of Medicine, King Saud Bin Abdulaziz University for Health Sciences, Riyadh, SAU; 5 Department of Orthopedics, Al-Imam Abdulrahman Al Faisal Hospital, First Cluster, Ministry of Health, Riyadh, SAU

**Keywords:** recurrence, radiotherapy, surgical synovectomy, arthroscopic synovectomy, pigmented villonodular synovitis

## Abstract

Introduction

Pigmented villonodular synovitis (PVNS) is a proliferative disorder that affects synovial joints. PVNS, also known as tenosynovial giant cell tumor (TGCT), is a broad term that refers to a variety of diseases, both localized and diffuse. The current study aims to describe the outcomes of PVNS in patients in King Abdulaziz Medical City (KAMC) Riyadh, Saudi Arabia especially after surgical resection, highlighting radiological and pathological evidence of disease recurrence and symptom progression. The study is also concerned with comparison between patients who had adjuvant radiotherapy versus patients who underwent surgery only in addition to comparison between patients who underwent arthroscopic surgery versus those who underwent open surgery.

Objective

Pigmented villonodular synovitis is a disorder that occurs in the synovial joints with the highest incidence rates in the third and fourth decades. PVNS is categorized into localized and diffuse. The current study aims at defining the recurrence rate and outcomes of PVNS in patients in KAMC between 2008 and 2019.

Design

A retrospective study that comprises patients with proven PVNS of the knee, hip, and elbow for a minimum follow-up duration of two years. Data was collected from the KAMC database, National Guard Health Affairs, Riyadh, Saudi Arabia.

Results

Nearly a third of patients reported a prior history of trauma. The knee joint was most frequently involved followed by the ankle followed by the hip. Diffuse PVNS was more common than localized disease with more affection on the right side. The most common presenting symptoms were pain and swelling followed by decreased range of motion (ROM). About one-fifth of patients had undergone prior surgery. 30.8% received adjuvant radiotherapy. Post-operative stiffness or decreased ROM were noted in 23.1% of cases. Half of the patients received follow-up for more than 24 months. Overall recurrence rate was 19.2%. Recurrence rates were significantly higher in patients with left-sided lesions and those with prior surgery but it did not reach statistical significance. Non-statistically significant trends for increased recurrences were also seen with older age, diffuse type PVNS, and knee involvement.

Conclusions

In summary, this study reports the outcomes of PVNS after surgical resection in patients in King Abdulaziz Medical City. The study showed recurrence rates being significantly higher in patients with left-sided lesions than those with prior surgery. Also, non-statistically significant trends for increased recurrences were also seen with older age, diffuse type PVNS, and knee involvement.

## Introduction

Pigmented villonodular synovitis (PVNS) is a proliferative disorder that affects synovial joints. In the United States, the annual incidence of PVNS is estimated to be 1.8/1000000, usually in young adults with the highest incidence noted in their third and fourth decades. However, it can also occur in the elderly and children [1]. PVNS, also known as tenosynovial giant cell tumor (TGCT), is a broad term that refers to a variety of diseases, both localized and diffuse. PVNS is an uncommon, benign, idiopathic synovial proliferative condition characterized by villous and/or nodular forms that have been seen in joints, tendon sheaths, and bursa [2]. PVNS is mostly a locally aggressive tumor. Generally, patients present with discomfort, edema, stiffness, and reduced range of motion in the affected joints [3]. PVNS is categorized according to the clinical presentation and radiological features into localized (L-PVNS) and diffuse (D-PVNS) [4]. L-PVNS is characterized by distinct nodular or pedunculated lesions, and the risk of recurrence is usually modest (0-15%) D-PVNS form is the most frequent type [5]. D-PVNS involves lesions within intra-articular soft tissue lining and exhibits more chronic picture in clinical presentation. Recurrence rates of D-PVNS post-medical intervention typically ranges from 9% to 46% [6]. The two types are different in terms of the clinical presentation, prognosis, and response to medical intervention [7]. Concerning the histopathology, PVNS is characterized by synovial hyperplasia and pigment deposition (hemosiderin) inside the joints and tendon sheaths in addition to the bursae. These lesions result in severe erosion that can affect structures [8,9]. Despite its benign histological appearance, PVNS can potentially progress into malignant form. Histological features of malignant PVNS types were characterized by (a) Nodular and solid infiltrative lesions; (b) Large, plump, round or oval cells with deep eosinophilic cytoplasm and indistinct borders; (c) Large nuclei with prominent nucleoli; and (d) Necrotic areas. Atypical mitoses were occasionally seen as well [4]. PVNS etiology is not clear with some literature suggesting a neoplastic process [10,11]. Numerous studies suggest a chronic inflammatory process [12]. Furthermore, a positive history of trauma has been reported in a huge percentage of PVNS patients [13].

The primary treatment options for PVNS are surgical resection via either open or arthroscopic synovectomy with radiation therapy [9]. A lot of studies showed that in order to achieve the best possible outcome in PVNS, synovectomy should be explored. Total synovectomy should be considered [8,14]. PVNS has a variable recurrence rate and may result in serious impairment of the joint function in the case of a long-term illness requiring multiple interventions leading to early secondary osteoarthritis and may necessitate joint replacement [15-17]. Adjuvant radiotherapy has been used in the literature and reportedly improves local control [9]. Previous studies address some factors that may play a role in the disease's recurrence including the history of previous surgery for PVNS of the same joint, diffuse type of disease (higher than localized), large joints, and adequacy of the synovectomy [18].

Some limitations are expected in the current study, such as the rare incidence of the disease and small sample size. There were several studies done on PVNS in the literature but none described the disease and its outcomes in Saudi Arabia.

The current study aims to describe the outcomes of PVNS in patients in King Abdulaziz Medical City (KAMC), Riyadh, Saudi Arabia especially after surgical resection, highlighting radiological and pathological evidence of disease recurrence and symptom progression. The study is also concerned with comparison between patients who had adjuvant radiotherapy versus patients who underwent surgery only in addition to comparison between patients who underwent arthroscopic surgery versus those who underwent open surgery.

## Materials and methods

Study design and subjects

The current study is a retrospective cohort study that includes all patients with proven PVNS of the knee, hip, and elbow for a minimum follow up duration of two years in King Abdulaziz Medical City. Collected data will include disease localization, therapeutic modalities and outcomes during a given period of time from the surgical database. The sample size includes all the cases in the period 2008-2019. Chart reviews were collected from the King Abdulaziz Medical City database, National Guard Health Affairs, Riyadh, Saudi Arabia.

Data collection, instruments used and measurements

A retrospective cohort study that includes reviewing of medical records of all patients with previously treated PVNS of the knee, hip, and elbow between 2008 and 2019. After institutional research ethics board approval of the study, data from all patients was collected. Their data was collected from paper charts and best-care systems, then recorded in a data collection form and finally entered into Excel spreadsheets (Microsoft, Redmond, WA, USA). Patients’ characteristics including gender, age at diagnosis, weight, height were recorded. Moreover, clinical presentation, affected joint side, symptom duration, co-morbidities, treatment strategy/choice of surgery and the need for total knee replacement (TKR) performed were recorded. Additionally, whether the patients received radiotherapy as an adjuvant treatment was recognized. PVNS were classified as diffuse or localized based on tumor extent on the magnetic resonance imaging (MRI) performed and confirmed by pathological report of biopsy taken intra-operative preoperatively.

Inclusion and exclusion criteria

The following inclusion criteria were used: (1) patients who were diagnosed with PVNS based on MRI and pathology report of biopsy taken, (2) patients who were followed up in King Fahad Hospital in King Abdulaziz Medical City, (3) patients who had previously untreated PVNS of the knee, (4) patients who are treated through excision with or without adjuvant radiation between the January 2008 and December 2018 and (5) patients who had been followed up for minimum of two years. Exclusion criteria in this study includes: (1) patients who were suspected to have PVNS but ruled out by final pathological diagnosis and (2) patients who had insufficient clinical data.

Data management and analysis plan 

Descriptive analysis was used to describe the data. Quantitative data were expressed as mean ± standard deviation (SD). All statistical tests were declared significant at p ≤ 0.05. Categorical data were expressed as frequency and percentage. Frequencies of different variables were compared with the recurrence in PVNS using chi-square test. For chi-square test p values ≤0.05, odds ratios (OR) with 95% confidence intervals (CI) were calculated as estimates of recurrence in PVNS against the studied factors. The collected data was analyzed using SPSS version 26.0 (IBM Corp., Armonk, NY, USA).

## Results

Demographic and baseline characteristics

The study included 26 patients undergoing surgical resection for PVNS. As summarized in Table 1, the mean age was 27.3 years and there was an equal distribution of males and females (50% each). The most common age group affected was 26-35 years (30.8%).

**Table 1:** Demographic information of patients included in the study

		N	N %	Mean (SD)
Sex	Female	13	50.0%	
	Male	13	50.0%	
Age in years				27.3 (11.8)
Age group	Less than 18	7	26.9%	
	18-25	5	19.2%	
	26-35	8	30.8%	
	More than 35	6	23.1%	

Clinical presentation and disease factors

Nearly one-third of patients (30.8%) reported a prior history of trauma. The knee joint was most frequently involved (76.9%), and diffuse PVNS (76.9%) was more common than localized disease (23.1%), with more affection of the right side as noted in 17 (65.4%) patients. The most common presenting symptoms were pain (80.8%) and swelling (53.8%) (Table 2).

**Table 2:** Clinical and pigmented villonodular synovitis characteristics of the study participants

		N	N %
History of trauma	Yes	8	30.8%
	No	18	69.2%
Presenting symptoms	Swelling only	4	15.4%
	Pain only	9	34.6%
	Painful swelling	8	30.8%
	Painful swelling with decreased range of motion	1	3.8%
	Swelling with decreased range of motion	1	3.8%
	Pain with decreased range of motion	3	11.5%
Frequency of individual symptoms	Pain	21	80.8%
	Swelling	14	53.8%
	Decreased range of motion	5	19.2%
Joint affected	Ankle	5	19.2%
	Hip	1	3.8%
	Knee	20	76.9%
Affected side	Left	9	34.6%
	Right	17	65.4%
Pigmented villonodular synovitis type	Diffuse	20	76.9%
	Localized	6	23.1%

Treatment details and outcomes

As shown in Table 3, most patients were treated with combined anterior and posterior surgical approaches (76.9%). For the anterior approach arthroscopic synocevectomy was performed and the posterior approach open synovectomy was performed. About one-fifth of patients (19.2%) had undergone prior surgery, with the first resection performed by an ortho-oncologist in 84.6% of the cases, while 30.8% received adjuvant radiotherapy. Post-operative stiffness or decreased range of motion (ROM) was noted in 23.1% of cases. Half of the patients received follow-up for more than 24 months, and the overall recurrence rate was 19.2%. 

**Table 3:** Management details and outcomes of patients included in the study

		N	N %
Surgical approach	Anterior	3	11.5%
	Anterior & posterior	20	76.9%
	Medial & lateral	1	3.8%
	Open	2	7.7%
Previous surgery	Yes	5	19.2%
	No	21	80.8%
Adjuvant radiotherapy	Yes	8	30.8%
	No	18	69.2%
First resection done by ortho-oncologist	Yes	22	84.6%
	No	4	15.4%
Post-operative complication	Stiffness/decreased range of motion	6	23.1%
	None	20	76.9%
Follow-up duration in months	Less than 6	6	23.1%
	6-12	3	11.5%
	12-24	4	15.4%
	More than 24	13	50.0%
Recurrence	Yes	5	19.2%
	No	21	80.8%

Analysis of risk factors for recurrence

As summarized in Table 4, recurrence rates were significantly higher in patients with left-sided lesions (80% vs 20%, p=0.034) and those with prior surgery (40% vs 14.3%, p=0.236) but it did not reach statistical significance. In addition, non-statistically significant trends for increased recurrences were also seen with older age, diffuse type PVNS, and knee involvement.

**Table 4:** Difference in recurrence rates according to demographic, clinical, and management details in patients included in the study

		Recurrence				
		Yes (n = 5)		No (n = 21)		p-value
		N	N %	N	N %	
Sex	Female	3	60.0%	10	47.6%	1.0^β^
	Male	2	40.0%	11	52.4%	
Age group	Less than 18	1	20.0%	6	28.6%	0.464^¥^
	18-25	1	20.0%	4	19.0%	
	26-35	3	60.0%	5	23.8%	
	More than 35	0	0.0%	6	28.6%	
Pigmented villonodular synovitis type	Diffuse	5	100.0%	15	71.4%	0.298^β^
	Localized	0	0.0%	6	28.6%	
Joint affected	Ankle	1	20.0%	4	19.0%	1.0^¥^
	Hip	0	0.0%	1	4.8%	
	Knee	4	80.0%	16	76.2%	
Affected side	Left	4	80.0%	5	23.8%	0.034*^β^
	Right	1	20.0%	16	76.2%	
Surgical approach	Anterior	1	20.0%	2	9.5%	0.763^¥^
	Anterior & posterior	4	80.0%	16	76.2%	
	Medial & lateral	0	0.0%	1	4.8%	
	Open	0	0.0%	2	9.5%	
Previous surgery	Yes	2	40.0%	3	14.3%	0.236^β^
	No	3	60.0%	18	85.7%	
Adjuvant radiotherapy	Yes	2	40.0%	6	28.6%	0.628^β^
	No	3	60.0%	15	71.4%	
First resection done by ortho-oncologist	Yes	4	80.0%	18	85.7%	1.0^β^
	No	1	20.0%	3	14.3%	
Post-operative complication	Yes	2	40.0%	4	19.0%	0.558^β^
	No	3	60.0%	17	81.0%	

## Discussion

Mostly, PVNS is a locally aggressive non-malignant tumor where patients present with discomfort, edema, stiffness, and decreased range of motion in the affected joints [3]. Most patients are successfully treated surgically where about 90% of patients with L-PVNS disease can be successfully treated with a simple excision [19]. On the other hand, D-PVNS is a benign but aggressive neoplastic inflammatory illness that causes severe morbidity and poor function in individuals [20]. Although some diffuse illness can be resected with a small amount of morbidity, the recurrence rate is estimated to be around 50% [21].

The aim of the present study is to characterize the outcomes of PVNS after surgical resection in patients in KAMC. The study focused on radiological and pathological evidence of disease recurrence and symptom progression. In addition, the study clarified post-operative symptoms, recurrence rates and risk factors for recurrence.

In the study, 26 patients were included with mean age of 27.3 years with equal distribution among males and females (50% each), compared to another study with a mean age of 33 and a 5:3 male-to-female ratio [22]. 23.1% of the cases that underwent resection of PVNS experienced post-operative stiffness or decreased ROM. Similarly noted by Casp et al. in which patients diagnosed with PVNS of the knee who underwent total knee arthroplasty reported higher rates of stiffness [23]. Many factors may contribute to post-operative stiffness and decreased ROM. The extent and invasiveness of the surgical procedure may play a role. PVNS can invade adjacent tissues leading to either extensive synovectomy or even joint resection in severe cases. These procedures can cause trauma to the neighboring structures, which results in limited joint mobility [24].

Overall recurrence rates in our study were 19.2%. Recurrence poses a major concern when it comes to PVNS, with overall recurrence rates being reported at 20%, similar to our findings [25]. It is crucial to note that the overall recurrence rate of 19.2% as observed in our study indicates a significant risk of PVNS recurrence. Therefore, regular follow-up and surveillance are critical for patients who have undergone surgical treatment for PVNS. The presented study revealed diffuse-type PVNS as a risk factor; however, it did not reach a statistically significant trend. Supporting our finding of increased recurrence in diffuse type, a study by Sharma and Cheng [7] reported a higher risk of recurrence in diffuse type and subtotal arthroscopic synovectomy. The diffuse type of PVNS encompasses a larger area of synovial tissue, which may result in the need for more extensive surgical approaches. However, even with aggressive synovectomy, complete removal may be difficult to achieve due to the infiltrative nature of the lesion. As a result, residual PVNS cells can remain, contributing to an increased risk of recurrence. In line with our work, among the evaluated factors, knee involvement showed a non-statistically significant trend towards increased recurrence of the knee in PVNS. Chiari et al. [26] reported a greater recurrence in larger joints as opposed to smaller joints. This is mainly due to the knee's extensive characteristics and complex anatomy resulting in difficulty in performing a complete total synovectomy. The knee joint is one of the most commonly affected sites in PVNS [27]. As a result of this difficulty in attaining a complete total synovectomy in the knee joint, it may play a role in residual disease and future recurrences.

The main limitations of the current study include small sample size of patients and the retrospective nature. However, this disease is infrequent and the number of instances is not large.

## Conclusions

In summary, this study describes the outcomes of PVNS after surgical resection in patients in KAMC. The study showed recurrence rates being significantly higher in patients with left-sided lesions than those with prior surgery. Also, non-statistically significant trends for increased recurrences were also seen with older age, diffuse type PVNS, and knee involvement.
